# Supplementary material for: Pan T cells, Helper T cells, and Regulatory T cells are Associated with Negative Symptoms in Persons with Anti-Gliadin Antibody Positive Schizophrenia and Related Disorders
Source: medRxiv. 2025 Feb 25:2025.02.24.25322815. Preprint. [Version 1] doi: 10.1101/2025.02.24.25322815 (PMC11888510; doi:10.1101/2025.02.24.25322815)
Supplement: 1 [file NIHPP2025.02.24.25322815V1-supplement-1.pdf]

medRxiv preprint doi: https://doi.org/10.1101/2025.02.24.25322815; this version posted February 25, 2025. The copyright holder for this preprint (which was not certified by peer review) is the author/funder, who has granted medRxiv a license to display the preprint in perpetuity.
It is made available under a CC-BY 4.0 International license .

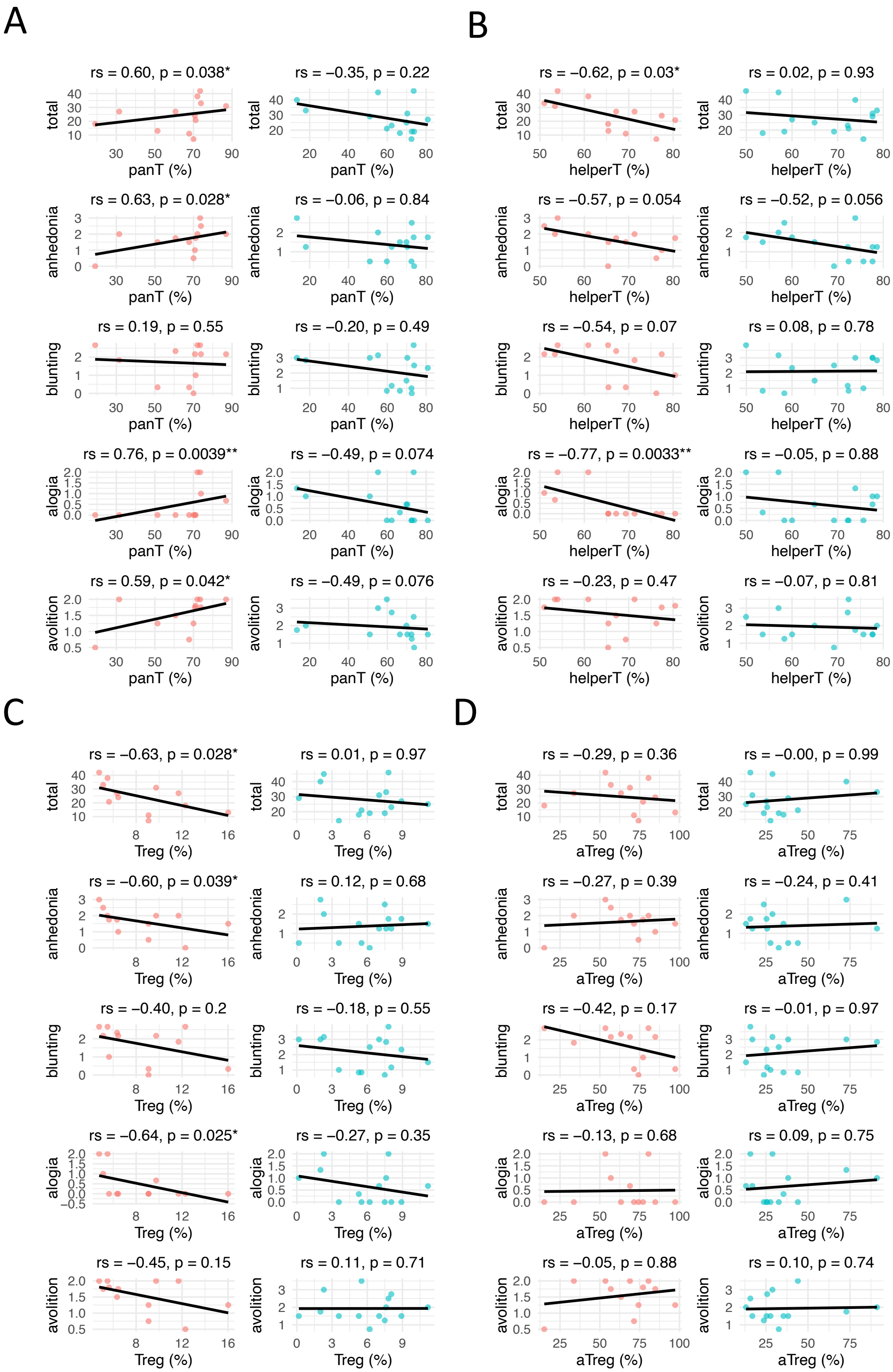

Figure S1: T cells correlate with negative symptoms in positive but not negative AGA-IgG schizophrenia. Spearman correlation coefficients (Spearman rs) were calculated with corresponding p values determining statistical significance. Scatterplots of each data point and a line of best fit corresponding with the correlation coefficient were generated in RStudio. A : Correlations between pan-T cells and negative symptoms. B: Correlations between helper T cells and negative symptoms. C: Correlations between Tregs and negative symptoms. D: Correlations between aTregs and negative symptoms. Abbreviations: panT (pan T cell) , defined as CD3+ T cells, helperT (helper T cell), defined as CD3+CD4+ T cells**,** Treg defined as CD3+CD4+CD25+Foxp3+ T cells, and aTregs defined as CD3+CD4+CD25+Foxp3+CD45RA- T cells. * indicates $p < 0.05$; ** indicates $p < 0.01$.
